# A microRNA gene expression signature predicts response to erlotinib in epithelial cancer cell lines and targets EMT

**DOI:** 10.1038/bjc.2011.465

**Published:** 2011-11-01

**Authors:** J L Bryant, J Britson, J M Balko, M Willian, R Timmons, A Frolov, E P Black

**Affiliations:** 1 Department of Pharmaceutical Sciences, College of Pharmacy, 343 Bio-Pharm Complex, University of Kentucky, Lexington, KY 40536-0082, USA; 2 University of Alabama-Birmingham, Comprehensive Cancer Center-Laboratory of Surgical, Oncology, Birmingham, AL 35294, USA; 3 Markey Cancer Center, Comprehensive Cancer Center-Laboratory of Surgical, Oncology, University of Kentucky, Lexington, KY 40536-0082, USA

**Keywords:** biomarker, targeted therapy, invasion, metastasis

## Abstract

**Background:**

Treatment with epidermal growth factor receptor (EGFR) inhibitors can result in clinical response in non-small-cell lung cancer (NSCLC) and pancreatic ductal adenocarcinoma (PDAC) for some unselected patients. EGFR and KRAS mutation status, amplification of EGFR, or gene expression predictors of response can forecast sensitivity to EGFR inhibition.

**Methods:**

Using an NSCLC cell line model system, we identified and characterised microRNA (miRNA) gene expression that predicts response to EGFR inhibition.

**Results:**

Expression of 13 miRNA genes predicts response to EGFR inhibition in cancer cell lines and tumours, and discriminates primary from metastatic tumours. Signature genes target proteins that are enriched for epithelial-to-mesenchymal transition (EMT) genes. Epithelial-to-mesenchymal transition predicts EGFR inhibitor resistance and metastatic behaviour. The EMT transcription factor, ZEB1, shows altered expression in erlotinib-sensitive NSCLC and PDAC, where many signature miRNA genes are upregulated. Ectopic expression of mir-200c alters expression of EMT proteins, sensitivity to erlotinib, and migration in lung cells. Treatment with TGF*β*1 changes expression of signature miRNA and EMT proteins and modulates migration in lung cells.

**Conclusion:**

From these data, we hypothesise that the tumour microenvironment elicits TGF*β*1 and stimulates a miRNA gene expression program that induces resistance to anti-EGFR therapy and drives lung tumour cells to EMT, invasion, and metastasis.

Epidermal growth factor receptor (EGFR) dependency has been observed among several tumour types, including non-small-cell lung cancer (NSCLC), pancreatic ductal adenocarcinoma (PDAC), and colorectal cancers. When utilised in unselected NSCLC patients, treatment with EGFR inhibitors results in modest response rates (Pao and Miller, 2005). Although both clinical and biological markers are associated with response to EGFR inhibitors, attempts to improve predictive accuracy using single-gene biomarkers such as EGFR mutation/amplification status, or KRAS gain-of-function mutations have been marginally successful, depending on the tumour type. Efforts to improve predictive capacity using multivariate biomarkers, such as gene expression predictors of response (GEPR) for erlotinib in NSCLC, have been described by this laboratory and others (Balko *et al*, 2006; Coldren *et al*, 2006). We hypothesised that microRNA (miRNA) expression patterns in cell lines with varying response to EGFR inhibition could provide biological insight to the mechanism of sensitivity and function as biomarkers of response to therapy.

MicroRNAs are a class of regulatory RNAs responsible for post-transcriptional gene silencing by either degrading the target mRNA or preventing its translation (Bartel, 2004; Filipowicz *et al*, 2008). MicroRNA expression is crucial for the regulation of cell processes necessary for development, differentiation, growth, and survival (Alvarez-Garcia and Miska, 2005; Hagen and Lai, 2008). Expression profiling of miRNA revealed that these genes are deregulated in tumours and can function as either tumour suppressors or oncogenes, depending on cellular context and mRNA targets (Lu *et al*, 2005; Wiemer, 2007). The function of miRNA in tumourigenesis may encompass the transition to metastatic disease or response to therapy as previously reported for the mir-200 family of miRNA (Ceppi *et al*, 2010; Mongroo and Rustgi, 2010).

We identified an miRNA gene expression signature of sensitivity to the EGFR inhibitor, erlotinib, in an NSCLC cell line model system. We hypothesised that members of the signature mediate both epithelial-to-mesenchymal transition (EMT) and response to erlotinib. We tested whether this signature might predict sensitivity to EGFR inhibition in other epithelial tumour types. Characterisation of the miRNA signature in NSCLC cells and bronchial epithelial cells revealed additional insight into the role of EMT in response to therapy.

## Materials and Methods

### Cell culture, western blotting, and expression vectors

Non-small cell lung carcinoma (NSCLC) and pancreatic ductal adenocarcinoma (PDAC) cell lines were purchased from ATCC (NSCLC) or were gifted (PDAC) by Andrey Frolov (University of Alabama-Birmingham). Both were cultured in RPMI 1640 media containing 10% fetal bovine serum for 48 h, harvested and analysed by quantitative real-time PCR or western blotting. Non-small-cell lung carcinoma cells were authenticated by STR testing. Beas2B cells were purchased from ATCC. Beas2B cells were maintained in ACL-4 media. For TGF*β*1 treatment, A549 cells, A549+mir200c shRNA cells, and Beas2B cells were plated and allowed to adhere. TGF*β*1 (2 ng ml^−1^) was then added directly to growth media for 3 days.

Whole-cell extracts from NSCLC and PDAC cells were evaluated for protein content by western blot. Specifically, media were removed and then cells were collected in phosphate-buffered saline and pelleted by centrifugation. Each pellet was resuspended in sample buffer and proteins were separated by gel electrophoresis. The gel was transferred to Immobilon membrane and probed with either ZEB1, E- or N-cadherin, Snail, pERK1/2, *β*-catenin, *α*-tubulin, or calnexin antibodies, each obtained from Cell Signaling Technologies (Beverly, MA, USA).

Ectoptic expression of mir-200c was carried out using an expression plasmid containing the mir-200c precursor expressed from an EF-1*α* promoter and a puromycin resistance marker (Cell Biolabs). A549 cells were transfected with the plasmid construct using Lipofectamine 2000 and selected with puromycin, producing a stable cell line.

Depletion of mir-200c was carried out by transducing Beas2B cells with a lentiviral expression vector (pTripZ) containing a short hairpin RNA (shRNA) sequence complimentary to mir-200c and a puromycin resistance gene (Systems Biosciences). Cells were infected and selected using puromycin, thereby creating a stable cell line.

### Sulforhodamine-B assay

Sulforhodamine-B assays measure proliferation and cell viability using total protein content as a metric. These assays were carried out as previously described following treatment with erlotinib (Balko *et al*, 2006).

### RNA extraction and analysis

Total RNA was prepared using the MirVana kit (Ambion) according to manufacturer's instructions. RNA was converted to cDNA by using the iScript cDNA Synthesis Kit (BioRad) for mRNA or using ABI cDNA synthesis reagents for specific miRNA messages (Applied Biosystems (ABI), Foster City, CA, USA). See below for ABI miRNA card methodology.

For single-gene expression measures, quantitative real-time PCR (qPCR) was carried out using TaqMan universal PCR master mix and gene-specific TaqMan gene expression probes (ABI). Human GAPDH was used as a control for mRNA expression, and U6 snRNA was the control used for miRNA expression. Samples were analysed in triplicate. Quantitative PCRs were run on a 7900HT Fast Real-Time PCR System (ABI). The resulting data were analysed by comparative *C*_t_ method using U6 (miRNA) and GAPDH (mRNA) as normalisation probes (Schmittgen and Livak, 2008).

### High-throughput miRNA analysis and signature identification

Total RNA from NSCLC or PDAC cell lines or tumour samples was prepared using the mirVana kit and reverse transcribed to cDNA. The cDNA was preamplified using ABI Megaplex primers (ABI), and the preamplified product was utilised for real-time PCR and subsequent hybridisation to ABI Taqman MicroRNA Array A cards (University of Kentucky AGTC core facility). Taqman MicroRNA array expression data were processed using SDS 2.3 and RQ Manager 1.2 Software (ABI). For analysis, genes that were undetectable were assigned a *C*_t_ value of 40, corresponding to the maximum cycle during the RT–PCR. Because some targets were not expressed, fold changes may be inflated in some instances. Samples were normalised by subtracting the average of the endogenous control *C*_t_ values across all samples (Δ*C*_t_). *C*_t_ values from four EGFR-inhibitor resistant cell lines (A549, UKY29, H460, and H1975) and four EGFR-inhibitor sensitive cell lines (H1650, H3255, H358, and PC9) were analysed by Student's *t*-test with *α*=0.05. Significantly changed probesets (38) were used to identify a predictive miRNA signature (*P*<0.1). The Δ*C*_t_ values for 38 signature miRNAs were used to train a predictive diagonal linear discriminant model (DLDA), according to our previously published methods (Balko *et al*, 2006). The discriminant function generates two scores for each unknown, according to the likelihood that the sample belongs to either the resistant or sensitive training data distribution. Samples falling below the identity line are considered resistant and those above the identity line are considered sensitive (Table 1). The 38 probes were reduced to 13 probes that best predicted erlotinib sensitivity, as determined by leave-on-out cross-validation and prediction of response using external validation data (see below). Ten thousand (10 000) 13-gene miRNA signatures were randomly generated from the training data using R and used to predict 16 unknown cases with the DLDA algorithm. The 13-gene miRNA signature significantly outperformed randomly generated signatures (9510/10 000 cases; adjusted *P*-value=0.049).

External expression data sets tested were derived from lung cancer cell lines (H2122, HCC327, H322, and H820), pancreatic cancer cell lines (Panc-1, MiaPaca, Aspc-1, and Bxpc3), or tumour samples from lung (primary tumours A–D; metastatic tumours U38-40) and colorectal cancer patients (metastatic tumour U41) treated with EGFR inhibitors that were collected under an institutional IRB-approved protocol.

Gene expression data (Δ*C*_t_ values) were used to cluster similarly expressed genes. Heatmaps were generated using GenePattern (Reich *et al*, 2006). Blue denotes low expression values, and red represents high expression.

MicroRNA genes were tested for mRNA targets using TargetScan, miRDB, and miRANDA, as each algorithm determines target binding differently. We selected targets determined by miRANDA/miSVR with scores less than −1.25 for further analysis. Potential targets for each miRNA were imported into Ingenuity Pathway Analysis (IPA) to assess pathway enrichment. Significantly counted pathways for each miRNA are shown in Table 2 and by network analysis (Supplementary Figure 1).

### Wound-healing assay

Beas2B cells or Beas2B cells transduced with a mir-200c shRNA lentivirus were plated and allowed to grow to a confluent monolayer. Cells were treated with 2 ng ml^−1^ TGF*β*1 24 h prior to the scratch. Cells were ‘wounded’ by scratching the monolayer with a pipet tip. The width of the wound was measured in 10 fields of view using brightfield microscopy (Zeiss AxioObserver, Axiovision Software) for 4 days and averaged. Data are presented as percentage of day 0 wound width.

## Results

### Identification and validation of the miRNA expression signature of response

We initiated these studies by identifying a signature of response to erlotinib using miRNA expression levels in NSCLC cell lines. In a previous publication, we separated a panel of NSCLC cell lines into sensitive and resistant to erlotinib treatment by measuring apoptosis after 48 h of treatment (Balko *et al*, 2006). Here, we measured the expression of 381 miRNA genes in the same resistant (A549, UKY-29, H460, and H1975) and sensitive (H1650, H3255, PC-9, and H358) cell lines using Taqman microRNA arrays.

**Figure 1:**
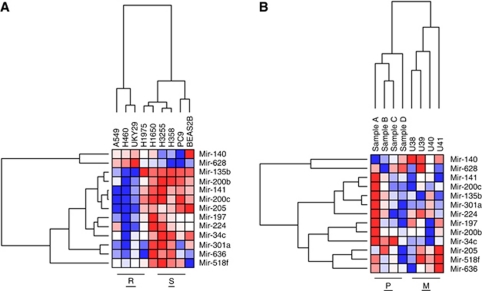
Hierarchical clustering of 13-gene microRNA signature. (**A**) Signature miRNA clustered by expression values and sample (lung cell lines) using GenePattern (Broad Institute) (**B**) Signature miRNA clustered by expression and sample (lung and colorectal tumours). Samples A–D are primary lung tumours (P) from patients treated with erlotinib. Samples U38–U40 are metastatic lung tumours (M) from patients treated with erlotinib and U41 is a metastatic colorectal tumour from a patient treated with the EGFR inhibitor, cetuximab.

Gene expression data were normalised and filtered, then a *t*-test was used to identify genes differentially expressed between erlotinib-sensitive and -resistant cell lines. A 13-gene miRNA signature was selected from 38 differentially expressed miRNA genes (*P*<0.1), because this signature was the most accurate of trained models in predicting response in validation data sets. The 13-gene signature miRNAs were clustered by relative expression levels and presented as a heatmap (Figure 1A). Eleven of the signature miRNAs are upregulated, and two miRNAs were found to be downregulated, in erlotinib-sensitive cells (S in Figure 1; H1650, H3255, H358, and PC9).

The 13-gene signature was used to develop a predictive algorithm of response to erlotinib utilising our previously published DLDA (Balko *et al*, 2006). A leave-one-out cross-validation assay was used to internally validate the predictor using training cell line data (resistant lines=A549, H460, H1975, and UKY29; sensitive lines=H1650, H3255, H358, and PC9). H1975 cells failed this validation. H1975 cells are genetically different compared with the other resistant lines in that they contain an EGFR mutation correlating with sensitivity and a second mutation that confers resistance (Gendreau *et al*, 2007; Figure 1A).

Further validation of the predictive accuracy of the selected 13-gene signature was carried out. First, the signature was compared with randomly selected 13-gene predictors for association with sensitivity to EGFR inhibitors using external validation data. We randomly selected 10 000 13-gene predictors from all 378 probes to test the accuracy of the selected signature. Our selected signature was significantly better than randomly selected signatures at predicting response (*P*=0.049). Next, expression data from NSCLC and PDAC cell lines with previously described erlotinib sensitivities were tested using the predictive signature (Eberhard *et al*, 2005; Tzeng *et al*, 2007). Finally, expression data from seven NSCLC and one colorectal cancer (primary and metastatic tumours), paraffin-embedded tumour samples from patients treated with EGFR inhibitors were also tested. These data indicate that with further validation the miRNA expression signature of erlotinib response may have clinical utility (Table 1).

Interestingly, we found that the 13-gene signature could not only predict response of patients to erlotinib, but could also discriminate primary (P) from metastatic (M) tumours, suggesting that the biological phenotypes underlying the signature were associated with both resistance to EGFR inhibition and metastatic behaviour (Figure 1B).

### Annotation of the 13-gene signature of response

**Figure 2:**
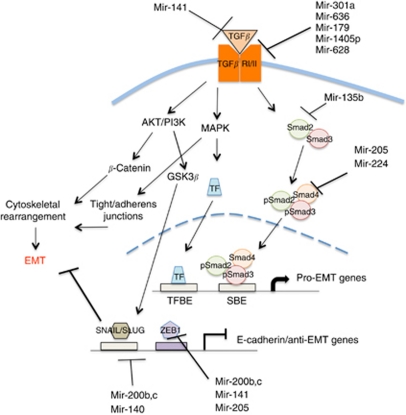
TGF*β* response pathway. Highlighted are targets of the microRNAs that are members of the response signature.

To dissect the biological contribution of signature miRNAs to erlotinib sensitivity and potentially to metastasis, we searched for potential target mRNAs of the miRNA genes in our predictive signature using TargetScan, miRDB, and miRANDA. Targets identified by miRANDA/miSVR with scores less than −1.25 were imported into IPA and assessed for gene ontology and/or signalling pathway membership. The most significantly counted canonical pathway is indicated for each miRNA along with the *P*-value of enrichment in Table 2, and relevant targets are noted in Figure 2.

Targets of the signature miRNAs were enriched for Wnt/*β*-catenin canonical signalling pathway (Table 2). Using highly enriched canonical pathways from each miRNA surveyed, networks of signalling events were constructed. The networks contain actual and inferred genes from the signature miRNA targets. The networks revealed that TGF*β*1 was a hub in networks formed by mir-140, -636, -301a, -224, and -200c genes (Supplementary Figure 1), even though the TGF*β*1 canonical signalling pathway was the top-enriched pathway in only the mir-636 network (Table 2). Signalling events important in EGFR biology were also evident, including MAPK and EGFR signalling pathways, but not as the most counted pathways.

Literature interrogation of signature miRNAs indicated that the expression cluster (Figure 1A) containing the mir-200 family, mir-205, mir-135b, and mir-141 target genes essential for the regulation of EMT (Burk *et al*, 2008; Park *et al*, 2008; Mongroo and Rustgi, 2010). Using these data, we decided to pursue the role of the 13-gene signature on TGF*β*1-induced EMT and erlotinib sensitivity.

### Biological characterisation of the signature members and EMT

Epithelial-to-mesenchymal transition regulator genes, such as E-cadherin and vimentin, are included in previously published signatures of response to EGFR inhibition (Yauch *et al*, 2005; Coldren *et al*, 2006; Thomson *et al*, 2005). Similarly, we identified ∼1500 genes with altered expression between erlotinib-sensitive and -resistant cells (Balko *et al*, 2006). Although EMT genes were not included in our 180-gene predictor of response, we re-evaluated the ∼1500 genes used to generate the 180-gene GEPR to identify other genes that may influence in EMT.

ZEB1/TCF8, a target of the mir-200 family of miRNA, was significantly downregulated in erlotinib-sensitive cells (*P*=1.51801E−17; Balko *et al*, 2006). E-cadherin, a target repressed by ZEB1, was correspondingly upregulated in the same cells consistent with the observations of others (Yauch *et al*, 2005; Coldren *et al*, 2006). Interestingly, we found that the mir-200 family of miRNA is upregulated in erlotinib-sensitive cells providing a possible explanation for ZEB1 loss in these cells (Figure 1A).

**Figure 3:**
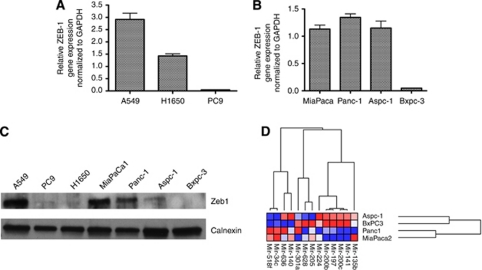
ZEB1 is expressed in erlotinib-resistant lung and pancreatic cancer cell lines. (**A**) ZEB1 mRNA expression in lung cancer cell lines: A549, H1650, and PC9. (**B**) ZEB1 mRNA expression in pancreatic cancer cell lines: MiaPaca, Panc-1, Aspc-1, and Bxpc-3. (**C**) ZEB1 protein expression in lung and pancreatic cancer cell lines. Calnexin serves as a loading control. (**D**) Expression of 13-gene miRNA signature in pancreatic cell lines, clustered by expression and sample as in Figure 1.

To validate the microarray expression data, A549 (EGFR wt), PC9 (EGFR delE746-A750), and H1650 (EGFR delE746-A750) were assayed for expression of ZEB1. Expression was measured by qPCR in each cell line (Figure 3A). ZEB1 expression in A549 cells (erlotinib resistant) is two- to three-fold higher than in either of the two EGFR mutant, erlotinib-sensitive cells (H1650 and PC9), supporting the microarray analysis. However, ZEB1 protein is upregulated in A549, while H1650 and PC9 cells poorly express ZEB1 protein despite varying levels of mRNA (Figure 3C). These data indicate that miRNA targeting ZEB1 message may inhibit translation rather than promote degradation of the message.

To understand whether sensitivity to erlotinib and expression of EMT genes is mediated by similar mechanisms in pancreatic cancers, as in NSCLC, ZEB1 protein expression was also evaluated in the PDAC cell lines. We hypothesised that ZEB1 gene expression would be reduced in the erlotinib-sensitive cell lines (Aspc-1 and Bxpc-3) compared with the erlotinib-resistant lines (MiaPaCa and Panc-1). ZEB1 mRNA was highly expressed in three of the four lines, but was not significantly expressed in Bxpc-3 cells (Figure 3B). ZEB1 protein expression correlated with gene expression data in three of four cell lines. Importantly, in erlotinib-sensitive pancreatic cancer lines, ZEB1 protein levels were reduced (Figure 3C). These data indicate that miRNAs can inhibit translation or promote message degradation, and ZEB1 mRNA expression alone is not a reliable surrogate for ZEB1 protein levels. Figure 3D illustrates expression levels of the 13 signature miRNA genes demonstrating that erlotinib-sensitive pancreatic cancer cell lines have high expression of the mir-200 family similar to the NSCLC-sensitive cells.

Together, these data indicate that signature miRNAs highly expressed in erlotinib-sensitive cells can control expression of target genes involved in EMT induction. We will specifically explore the role of mir-200c, a representative member of the highly expressed cluster of signature miRNAs, in lung cell lines to determine what signals can modulate expression of the miRNA, influence induction of EMT, and impact response to erlotinib.

### Expression levels of mir-200 modulate both signature miRNA and EMT targets

**Figure 4:**
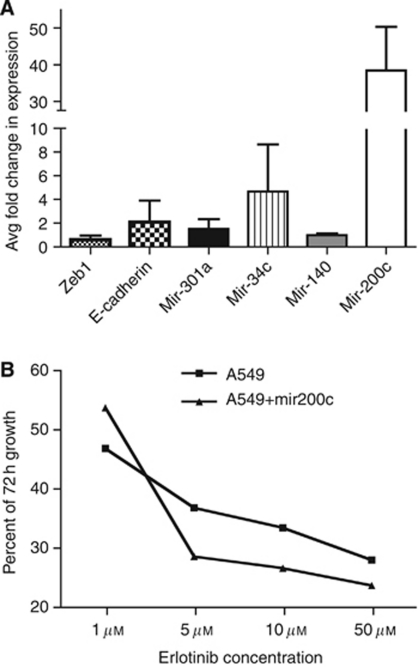
Ectopic expression of mir-200c alters expression of signature miRNA, EMT genes, and modulates sensitivity to erlotinib in A549 cells. (**A**) ZEB1, E-cadherin, mir-34c, mir-301a, and mir-140 levels were determined in A549 cells. The mir-200c precursor was expressed from a plasmid vector, and mir200c-expressing cells were selected with puromycin. Expression of the query genes was evaluated by qPCR in control and transfected cells. The data are the mean fold change, relative to control (A549 parent cells), of two independent experiments each evaluated in triplicate showing standard deviation (s.d.). (**B**) A549 and A549+mir200c-expressing cells were treated with erlotinib for 72 h in 0.1% serum-containing RPMI. Cell growth was measured by SRB assay in triplicate, averaged, and compared with 0 h controls. Cell growth at 72 h is plotted against erlotinib concentration.

We used erlotinib-resistant A549 lung adenocarcinoma cells for initial characterisation of the role of mir-200c in erlotinib sensitivity and EMT induction. A549 cells express low levels of mir-200c compared with erlotinib-sensitive NSCLC cells, such as PC9 (Figure 1A). We expressed mir-200c from a lentiviral expression vector in A549 cells and observed increased mir-200c message levels, no change in ZEB1 expression, and gene expression of E-cadherin relative to the parent cells (Figure 4A). We also investigated the expression of representative miRNA from each of the major expression clusters (Figure 1A) in A549 cells expressing ectopic mir-200c. Expression of mir-301a and -34c each increased, relative to parent A549 cells.

A549 cells are insensitive to erlotinib at concentrations that kill EGFR mutant cells. We asked whether ectopic expression of mir-200c, resulting in increased expression of a subset of the signature miRNAs, would increase sensitivity to erlotinib. Ceppi *et al* (2010) previously showed that introduction of mir-200c into H1299 cells restored sensitivity to cetuximab, also an EGFR inhibitor. Sensitivity to erlotinib was tested using concentrations from 1 to 50 *μ*M in A549 and A549+mir200c cells. Ectopic expression of mir-200c modestly increased the sensitivity of A549 to erlotinib, similar to results observed by others (Ceppi *et al*, 2010; Figure 4B).

ZEB1 expression, induction of EMT, and erlotinib sensitivity are likely controlled by the combined activity of many factors, including the signature miRNA reported here. We furthered these observations by evaluating expression of mir-200c and proteins involved in the induction of EMT following treatment with TGF*β*1, an EMT inducer, because several signature miRNAs target members of the TGF*β* response pathway (Figure 2, Supplementary Figure 1).

### TGF*β*1 controls the expression of mir-200c

Ingenuity Pathway Analysis software revealed that TGF*β* signalling pathway members are targets of signature miRNAs (Figure 2, Supplementary Figure 1). TGF*β*1 can initiate EMT, and EMT is a hallmark of tumour invasion and metastasis. TGF*β*1 signalling may also contribute to erlotinib response in lung cancers (Yao *et al*, 2010). Therefore, we asked whether the induction of EMT by TGF*β*1 is mediated by differential regulation of signature miRNA expression, especially that of mir-200c.

Epithelial-to-mesenchymal transition is characterised by increased expression of mesenchymal markers, such as ZEB1 and N-cadherin, while losing epithelial markers such as E-cadherin. A549 cells demonstrate a mesenchymal phenotype, but express both Zeb1 and E-cadherin. Because EMT is a plastic state, allowing cells to vary expression of EMT-specific proteins, cells grown in two dimensions may not commit to either epithelial or mesenchymal states. The impact of TGF*β*1 treatment in both A549 cells and mir-200c-expressing A549 cells was evaluated by expression of RNAs and proteins important for EMT (Adam *et al*, 2009; Gibbons *et al*, 2009).

**Figure 5:**
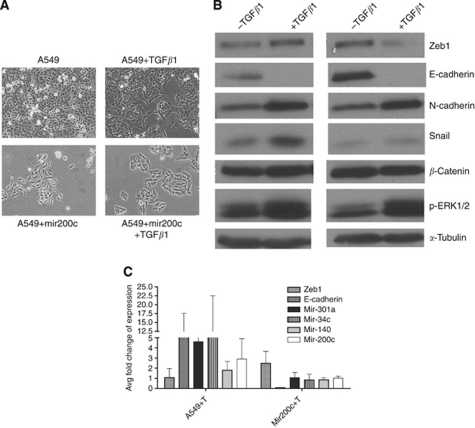
TGF*β*1 treatment has differential effects on EMT in A549 cells compared with ectopic mir-200c expression. (**A**–**C**) A549 or A549+mir200c cells were treated for 3 days with TGF*β*1 (2 ng ml^−1^). (**A**) Cellular morphology is altered in the presence TGF*β*1 in both cell lines. (**B**) Proteins involved in EMT are differentially expressed in the presence of TGF*β*1 in A549 and A549+mir200c-expressing cells. ZEB1, E-cadherin, *β*-catenin, Snail, and pERK1/2 expression was determined by western analysis. *α*-Tubulin serves as a loading control. (**C**) Expression of EMT effector mRNA and a panel of signature miRNAs are differentially expressed in A549 and A549+mir200c expressing. Expression of ZEB1, E-cadherin, mir-200c, mir-34c, mir-301a, and mir-140 genes were evaluated by qPCR in control and mir-200c-expressing cells. The data are mean fold change, relative to control (untreated A549 cells), of two independent experiments each evaluated in triplicate. Bars show standard deviation (s.d.).

In both A549 and A549+mir200c cells, TGF*β*1 treatment changed cellular morphology (Figure 5A). TGF*β*1 predictably reduced E-cadherin and increased ZEB1, N-cadherin, and Snail in A549 cells (Figure 5B). Unexpectedly, all miRNAs tested demonstrated increased expression relative to untreated cells (Figure 5C). Of the miRNAs tested, we expected mir-140 to be the only miRNA upregulated, as it does not obviously link to EMT induction and is poorly expressed in erlotinib-sensitive cells. In A549+mir200c cells after treatment, both ZEB1 and Snail proteins are poorly expressed, and the miRNAs tested are expressed at the same level as untreated parental A549 cells. Thus, TGF*β*1 treatment counters the ectopic expression of mir-200c, but cannot fully induce EMT (poor Snail expression and decreased ZEB1) and does not alter response to erlotinib (Supplementary Figure 2). These data indicate that mir-200c and TGF*β*1 treatment have independent effects on EMT induction.

To evaluate the influence of TGF*β*1 treatment on signature miRNA expression, the promoter region of each miRNA was evaluated for TGF*β*1-responsive elements using ChipMAPPER (Marinescu *et al*, 2005). mir-200c, -141, -34c, and -301a promoters contain SMAD3/4 binding sites, indicating probable responsiveness to TGF*β*1 signalling. Further experiments will be required to confirm this observation.

### TGF*β*1 or reduction of mir-200c levels accelerates wound healing

Beas2B cells, immortalised bronchial epithelial cells, have not undergone complete EMT and express high levels of both mir-200c and ZEB1. We utilised a wound-healing assay to evaluate the migration response, as an *in vitro* surrogate of metastasis, of Beas2B cells in the presence of TGF*β*1 treatment and/or reduction in mir-200c expression.

**Figure 6:**
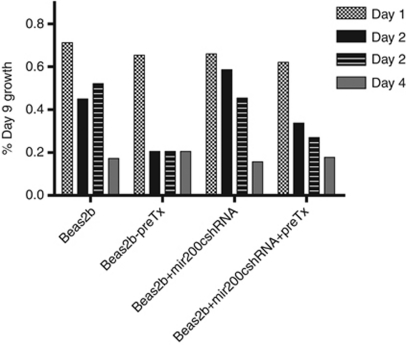
Treatment with TGF*β*1 and abrogation of mir-200c accelerates wound healing in Beas2B cells. Beas2B cells were transduced with a mir-200c shRNA lentivirus or mock infected. Both cell lines (Beas2B or Beas2B-anti-mir200c) were pre-treated for 24 h with TGF*β*1 (2 ng ml^−1^) then scratched and treatment was continued. The wound was measured in 10 fields of view each day for 4 days or until the wound closed to calculate standard deviation. Closure is expressed as percentage of day 0.

Beas2B cells were transduced with a lentiviral vector expressing an shRNA) to reduce mir-200c expression. Short hairpin RNA-transduced and control Beas2B cells were grown to a monolayer and scratched. Both mock and transduced cells were pre-treated with TGF*β*1 1 day prior to the scratch, and treatment continued throughout the experiment. Reduction in mir-200c expression accelerated wound healing compared with mock-transduced, parental Beas2B cells. The healing phenotype in both conditions is accelerated with TGF*β*1 treatment (Figure 6). These data indicate that TGF*β*1 and mir-200c participate in complementary pathways for migration in lung cells.

## Discussion

Molecular determinants of response to EGFR inhibitors have been extensively explored and range from single gene to multi-gene biomarkers (Yauch *et al*, 2005; Balko *et al*, 2006; Frederick *et al*, 2007; Haddad *et al*, 2009). Moreover, predictors of response to EGFR inhibitors do not necessarily depend on the expression of the target gene, EGFR. Recent reports have underscored the impact of EMT in the sensitivity of solid tumours to EGFR inhibitors such as erlotinib (Yauch *et al*, 2005; Frederick *et al*, 2007; Black *et al*, 2008; Haddad *et al*, 2009).

Here we report that sensitivity to the EGFR inhibitor, erlotinib, can be predicted by a 13-gene miRNA signature identified using NSCLC cell line expression data. Notably, the 13-gene signature was able to separate primary from metastatic tumour samples using unsupervised clustering methods. Ontological annotation of the 13 signature genes and their potential targets revealed enrichment in components of EMT, including Wnt pathway signalling, which may partially explain the ability of the signature to segregate metastatic tumours (Pacheco-Pinedo *et al*, 2011). We investigated the contribution of the 13 miRNA genes to erlotinib response and induction of EMT.

Annotation of the potential target genes of the member miRNA of the signature using multiple web-based tools revealed that the major cluster of commonly expressed miRNA genes (mir-200b, -200c, -135b, -141, and -205) can control expression of proteins involved in the induction of EMT. This cluster of miRNAs is highly expressed in erlotinib-sensitive cancer cells and Beas2B cells (Figure 2). Normally, EMT is a mechanism of cellular plasticity that is important for development, but is hijacked in tumour metastasis (Polyak *et al*, 2009). A double-negative feedback loop involving ZEB transcription factors and mir-200 family of genes has been described and explored by many groups (Burk *et al*, 2008; Park *et al*, 2008). Increased expression of mir-200 should reduce ZEB1 expression, induce E-cadherin expression, and reduce the invasive character of cells resulting in a more epithelial phenotype (Patton *et al*, 1991).

Non-small-cell lung cancer cell lines resistant to erlotinib often display EMT features (Yauch *et al*, 2005). We assessed EMT status using NSCLC and PDAC lines with known response to erlotinib, concentrating on the ZEB1–mir-200c axis. Therefore, measuring EMT induction in these cells allowed us to correlate erlotinib response with EMT by assessing levels of mir-200 and target, ZEB1. We found that levels of ZEB1 protein inversely correlated with expression of the mir-200 family of miRNAs, but levels of ZEB1 RNA varied in both NSCLC and pancreatic cancer cells.

We went on to utilise two lung cell lines to assess EMT induction in response to either TGF*β*1 or mir-200c: Beas2B (immortalised bronchial epithelial cells) and A549 (lung adenocarcinoma cells). Although both are resistant to EGFR inhibition, the Beas2B cells demonstrate a miRNA gene expression profile similar to erlotinib-sensitive cells, specifically expression of mir-200c (Figure 1).

TGF*β*1 has both positive and negative effects on EMT and metastasis (Derynck *et al*, 2001; Tu *et al*, 2003; Wendt *et al*, 2011). We examined expression of EMT components in A549 cells after 3 days of TGF*β*1 treatment. Although A549 cells have some mesenchymal features, TGF*β*1 treatment induced both ZEB1 protein and mir-200c expression. Interestingly, TGF*β*1 treatment of A549 cells also induced E-cadherin mRNA and representative signature miRNA expression (Figure 3). These data support the assertions of Polyak and others that EMT is a plastic phenotype that can be pushed with molecular cues in both directions (Thiery and Sleeman, 2006; Polyak *et al*, 2009).

Recently, Ceppi *et al* (2010) observed that sensitivity to EGFR inhibition and the aggressive nature of a tumour is a function of the expression of mir-200c. We found that the 13-gene signature represents the least complex miRNA expression profile that can predict sensitivity to erlotinib; ie, mir-200c expression alone cannot predict sensitivity to erlotinib (data not shown). Further, ectopic expression of mir-200c only marginally improves sensitivity of A549 cells to erlotinib, as measured by cell proliferation (Figure 4). Thus, the contribution of an miRNA expression programme that predicts erlotinib sensitivity is more complex than simply the activity of the mir-200 family.

To address the role of mir-200c in response to erlotinib and induction of EMT, mir-200c expression was experimentally modulated in A549 and Beas2B cells. A549 cells, normally expressing low levels of mir-200c, were transduced with a mir-200c expression construct. mir-200c expression was increased 50-fold over the parent A549 cells (Figure 4A). Treatment of A549 or A549+mir200c cells with TGF*β*1 resulted in similar morphological changes (Figure 5A) and changes in protein expression (Figure 5B) with notable exceptions. A549+mir200c cells treated with TGF*β*1 demonstrated reduced expression of mir-200c (compare Figures 5C and 4A). ZEB1 and Snail protein levels were reduced even though ZEB1 mRNA increased (Figure 5C compared with 4A). In our hands, ectopic expression of mir-200c in A549 cells does not completely replicate the observations of Ceppi *et al* (2010), although the cell lines and EGFR inhibitors used were different. Specifically, re-introduction of mir-200c into A549 cells does not change morphology, modestly changes expression of members of the EMT network (E- and N-cadherin, ZEB1, or Snail), and slightly improves response to EGFR inhibition. Inexplicably, in A549 cells expressing ectopic mir-200c, TGF*β*1 treatment diminishes mir-200c, even below the levels of the parental cells, and induces ZEB1 mRNA but not protein. These observations are very similar to the phenotype of Aspc-1 pancreatic cells observed in Figure 3, suggesting that mir-200c and TGF*β*1 may have non-overlapping roles in the control of EMT. Further experimentation is necessary to dissect these phenotypes.

Beas2B cells were transduced with a mir-200c shRNA lentiviral construct, and then tested for migration in a wound-healing assay in the presence and absence of TGF*β*1. Reducing mir-200c levels using shRNA resulted in enhanced migration compared with parental Beas2B cells and even further accelerated when cells were pre-treated with TGF*β*1. This observation enforces the notion that TGF*β*1 and mir-200c serve complementary pathways in EMT induction.

Although this work only interrogated the mir-200c/ZEB1 axis, we noticed that other representative signature miRNAs have altered expression in the presence of TGF*β*1 and/or mir-200c expression. Future studies will elucidate the interplay of the TGF*β*1 signalling cascade with expression and targets of the EGFR inhibitor sensitivity miRNA signature.

Finally, we envision that by coupling our GEPR and miRNA predictor of response to EGFR inhibition a diagnostic may be possible that improves the accuracy of selecting patients with tumours that respond to EGFR inhibition. This could be a significant benefit to the treatment of patients, particularly those with pancreatic cancer with EGFR-dependent phenotypes, but no known biomarkers of EGFR inhibitor response (Tzeng *et al*, 2007).

## Figures and Tables

**Table 1:** Validation samples used to test the 13-gene miRNA predictor

**Cell lines/tumour**	**Actual sensitivity**	**Predicted sensitivity**
Lung-2122	Sensitive (S)	Resistant (R)
Lung-827	S	R
Lung-322	S	R
Lung-820	R	S
Pancreatic-Aspc1	S	S
Pancreatic-Bxpc1	S	S
Pancreatic-MiaPaca	R	R
Pancreatic-Panc1	R	R
Primary tumours-lung A	S	S
Primary tumours-lung B	R	R
Primary tumours-lung C	R	R
Primary tumours-lung D	R	R
Met-U38-lung	R	R
Met U39-lung	R	R
Met U40-lung	S	S
Met U41-colorectal	S	R

Eight cell lines and eight tumours were used to prepare RNA for generation of miRNA expression data. Actual sample sensitivity is noted in column 2, and predicted sensitivity is shown in column 3.

**Table 2:** Targets of the 13-gene miRNA signature were annotated for pathway membership using Ingenuity Pathway Analysis (IPA)

**MicroRNA (hsa-mir-x)**	**Top canonical pathway (IPA)**	***P*-value of enrichment (IPA)**
140-3p	Glycophospholipid biosynthesis	6.99E–02
628-5p	B-cell development	1.02E–02
518f	Antiproliferative role of the somatostatin receptor 2	3.89E–02
636	TGF*β* signalling	2.54E–03
301a	Wnt/*β* catenin	1.99E–03
34c	Serotonin receptor signalling	3.66E–02
224	Hypoxia signalling	1.19E–02
197	Role of stromal cells in rheumatoid arthritis	1.31E–02
205	Biosynthesis of steroids	1.89E–02
135b	Circadium rhythm	2.6E–02
200b	Wnt/GSK signalling	4E–03
200c	Wnt/*β* catenin	8.17E–03
141	Integrin signalling	11.8E–04

The most significantly enriched pathway is denoted for each miRNA along with the *P*-value of enrichment as calculated by IPA.
