# Supplementary figures and images for: A microRNA gene expression signature predicts response to erlotinib in epithelial cancer cell lines and targets EMT
Source: Br J Cancer. 2011 Nov 1;106(1):148–56. doi: 10.1038/bjc.2011.465 (PMC3251842; doi:10.1038/bjc.2011.465)

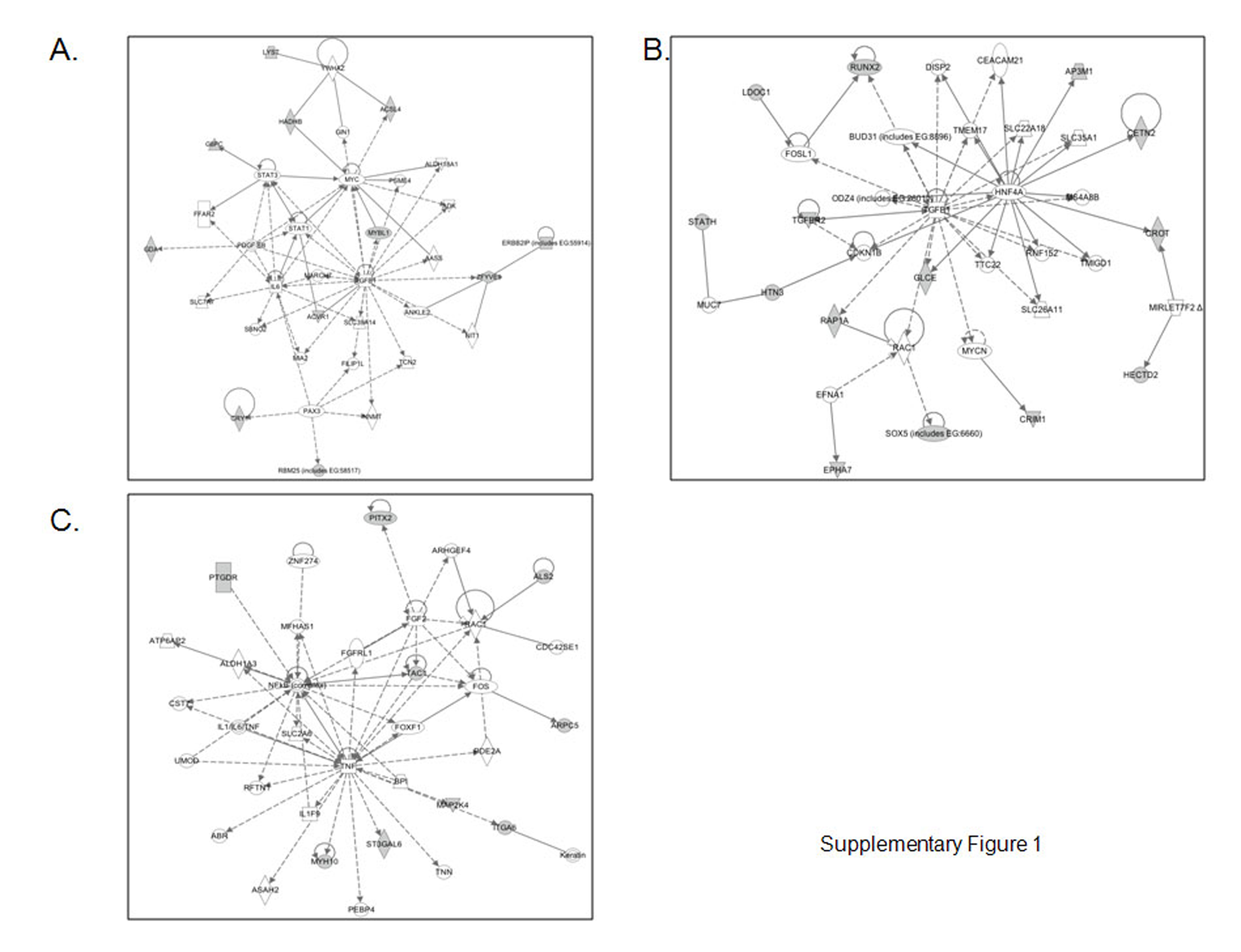

Supplement: Supplementary Figure 1 [file bjc2011465x1.tif]

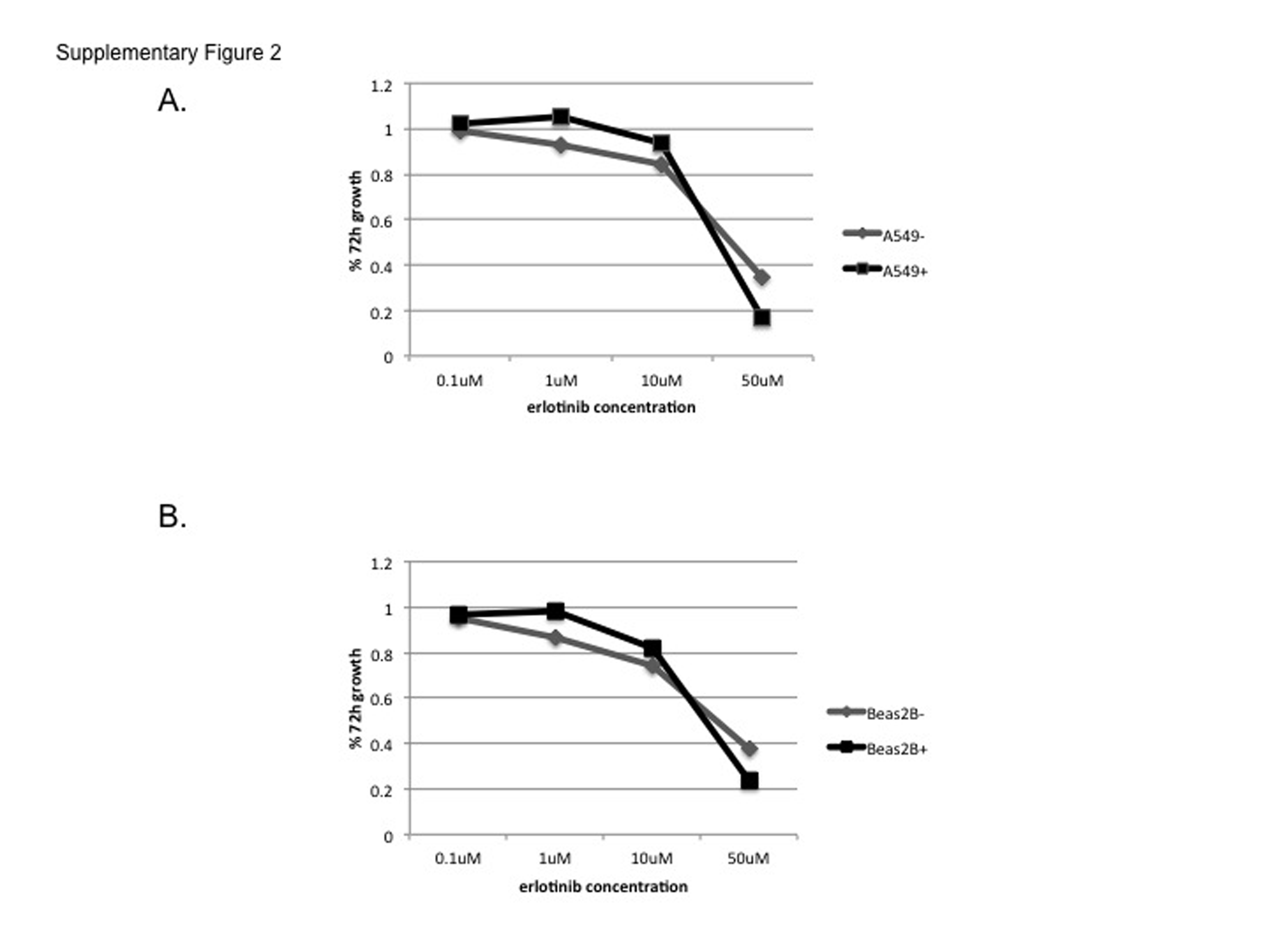

Supplement: Supplementary Figure 2 [file bjc2011465x2.tif]
