# Supplementary material for: A microRNA gene expression signature predicts response to erlotinib in epithelial cancer cell lines and targets EMT
Source: Br J Cancer. 2011 Nov 1;106(1):148–56. doi: 10.1038/bjc.2011.465 (PMC3251842; doi:10.1038/bjc.2011.465)
Supplement: Supplementary Figure Legends [file bjc2011465x3.doc]

Supplemental Files

Figure S1: Pathway networks were derived from Ingenuity Pathway analysis of target genes associated with specific signature miRNA. A. mir-301, B. mir-636, and C. mir-141. Specifically, targets from mir-301 and -636 have TGFβ1 as a hub while mir-141 can intersect TGFβ1 via Rac1 in the mir-636 network.

Figure S2: TGFβ1 treatment of A549 and Beas2b cells does not improve sensitivity to erlotinib.

A549 cells (A) and Beas2B cells (B) were treated with TGFβ1 for 3 days then passaged to 96-well dishes and were treated with erlotinib for 72h in 0.1% serum-containing RPMI. Cell growth was measured by SRB assay in triplicate, averaged, and compared with 0h controls. Cell growth at 72h is plotted against erlotinib concentration.
